# Congenital Dislocation of the Humerus Reduced after Sixteen Years

**Published:** 1841-05

**Authors:** 

**Affiliations:** Surgeon to the Hotel Dieu, of Poictiers


*Congenital Dislocation of the Humerus reduced after sixteen years.*—By M. Gaillard, Surgeon to the Hotel Dieu, of Poictiers. The following is an abstract of a report to the Royal Academy of Medicine, drawn up by MM. Cannet and Bouvier. They consider the case as very curious, and without a parallel in the history of surgery.

Mademoiselle L. B. a few days after birth presented a deformity of the left superior extremity with much pain during its movements. The elbow was thrown from the body, the forearm semiflexed, and the hand in a state of pronation. During her infancy she could not bring her elbow near the trunk, nor raise her hand higher than the chin. The limb was often painful during her early years; but latterly the pain had only come on when the limb had been fixed in one position. When M. Gaillard saw her in 1836, she was sixteen years of age. He found in the posterior part of the shoulder a projection formed by the head of the humerus, which was placed in the subspinous fossa of the scapula, above the middle part of the spine, which was curved by the pressure it had undergone. On moving the arm, the head of the bone was felt rolling under the fingers, and it appeared abraded from rubbing on an irregular surface. The hand could not be brought to a state of supination. The forearm could not be extended on account of the permanent contraction of the biceps. Elevation and rotation of the arm were impossible.

On the 5th of January, 1837, the patient was placed on a stool, a cushion was applied to the external border of the scapula and maintained by two cords passing from its extremities and attached to two fixed rings. The arm directed horizontally inwards was then submitted to the action of a weight of sixteen pounds. The weight was suspended at the end of a cord which passed through a fixed pully, and was attached to a bracelet fixed above the elbow. Time after time M. Gaillard increased the power of the traction, occasionally adding his efforts to the force exercised by the weights. The extension prolonged from twenty to twenty-five minutes was repeated on the 10th and 11th of January, each time bringing the head of the humerus nearer the glenoid cavity, and with no other inconvenience than a slight pain and swelling of the arm. On the 13th the head of the humerus was more moveable. Further traction was employed, the arm being always placed horizontally. The humerus yielded, glided over the scapula for the space of an inch and a half, and approached the glenoid cavity. M. Gaillard then seized the elbow, and by carrying it backwards and upwards he directed the head of the humerus downwards and forwards, then lowering the limb he felt the head pass under the acromial arch, and leap over a projection which appeared to belong to the articular cavity. The arm was now in contact with the trunk, it was sensibly lengthened, and all its movements could be executed with ease.

The dislocation, however, occurred again and again, and was as often reduced. The difficulty was to maintain the bone after reduction. This, however, was effected by careful bandaging. The patient suffered considerable pain at times, but was relieved by the application of leeches. From the first reduction to the entire cessation of pain, a period of more than two years elapsed; but the cure was then complete, the patient using the limb equally as well as the other.—*Bulletin de l'Acadamie Royale de Medecine. Maryland Med. and Surg. Jour. Vol.* 1. *No.* 4.

